# The genome sequence of the wall brown, *Lasiommata megera* (Linnaeus, 1767)

**DOI:** 10.12688/wellcomeopenres.18106.1

**Published:** 2022-09-12

**Authors:** Konrad Lohse, Wellcome Sanger Institute Tree of Life programme, Wellcome Sanger Institute Scientific Operations: DNA Pipelines collective, Tree of Life Core Informatics collective, Charlotte Wright, Darwin Tree of Life Consortium

**Affiliations:** 1 Institute of Evolutionary Biology, University of Edinburgh, Edinburgh, UK; 2 Wellcome Trust Sanger Institute, Hinxton, UK

**Keywords:** Lasiommata megera, wall brown, genome sequence, chromosomal, Lepidoptera

## Abstract

We present a genome assembly from an individual female *Lasiommata megera* (the wall brown; Arthropoda; Insecta; Lepidoptera; Nymphalidae). The genome sequence is 488 megabases in span. The majority of the assembly (99.97%) is scaffolded into 30 chromosomal pseudomolecules with the W and Z sex chromosomes assembled. The complete mitochondrial genome was also assembled and is 15.3 kilobases in length.

## Species taxonomy

Eukaryota; Metazoa; Ecdysozoa; Arthropoda; Hexapoda; Insecta; Pterygota; Neoptera; Endopterygota; Lepidoptera; Glossata; Ditrysia; Papilionoidea; Nymphalidae; Satyrinae; Satyrini; Parargina; *Lasiommata*; *Lasiommata megera* (Linnaeus, 1767) (NCBI:txid111917).

## Background

The wall brown, *Lasiommata megera* (Linnaeus 1767), is a widely distributed butterfly found across the Palearctic. This species inhabits open sunny places such as grasslands and sand dunes, and is known for basking on bare surfaces such as walls and rocks. Larvae feed on various grasses including false broom ( *B. sylvaticum),* tor-grass ( *Brachypodium pinnatum*) and bents ( *Agrostis* spp.). Forewings possess a single large eyespot, and hindwings contain four smaller eyespots, set against orange and brown markings. This species is generally bivoltine; adults can be found on the wing from May to October.

In the British Isles, this butterfly is widespread but scarce, with a higher density towards the coast. Since the 1970s, the wall brown has experienced a major decline in both abundance and occurrence in the British Isles, with an 87% decrease in abundance in southern Britain due to loss of colonies ( Fox *et al.*, 2015). The Wall has also experienced a significant decline in abundance across Europe based on the European Grassland Butterfly Indicator ( van Swaay *et al.*, 2013). One potential explanation for this decline is that warmer conditions due to climate change, may be triggering a third generation, resulting in a high mortality rate in autumn ( Van Dyck *et al.*, 2015). Other changes in the environment, such as nitrogen deposition, have also been implicated in their decline ( Klop *et al.*, 2015). The Wall has an estimated genome size of 381 Mb based on flow cytometry ( Mackintosh *et al.*, 2019).

## Genome sequence report

**Figure 1.**
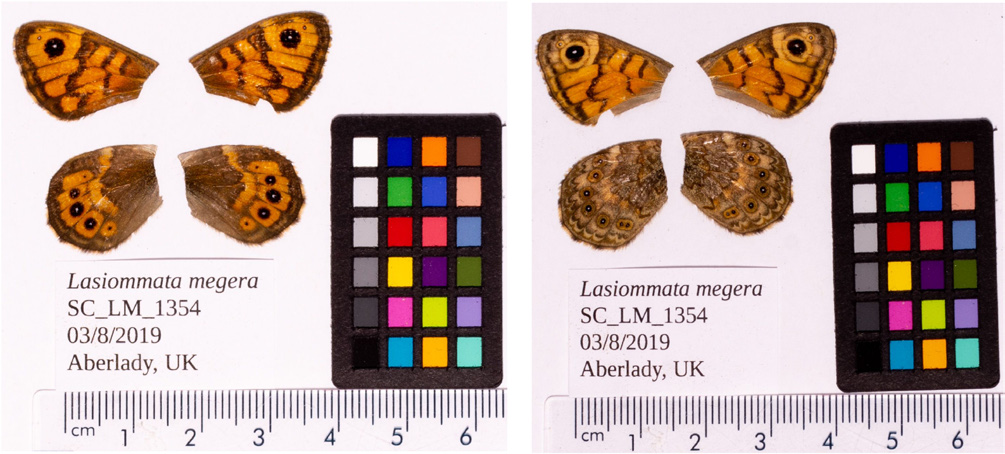
Fore and hind wings of the *Lasiommata megera* specimen from which the genome was sequenced. Dorsal (left) and ventral (right) surface view of wings from specimen SC_LM_1354 (ilLasMege1) from Aberlady Bay, Scotland, UK, used to generate Pacific Biosciences and 10X genomics data.

The genome was sequenced from a single female *L. megera* from Aberlady Bay, Scotland, UK ( Figure 1). A total of 45-fold coverage in Pacific Biosciences single-molecule HiFi long reads and 77-fold coverage in 10X Genomics read clouds were generated. Primary assembly contigs were scaffolded with chromosome conformation Hi-C data. Manual assembly curation corrected 17 missing/misjoins and removed 1 haplotypic duplication, reducing the assembly size by 0.33% and the scaffold number by 24.56%, and increasing the scaffold N50 by 0.002%.

**Figure 2.**
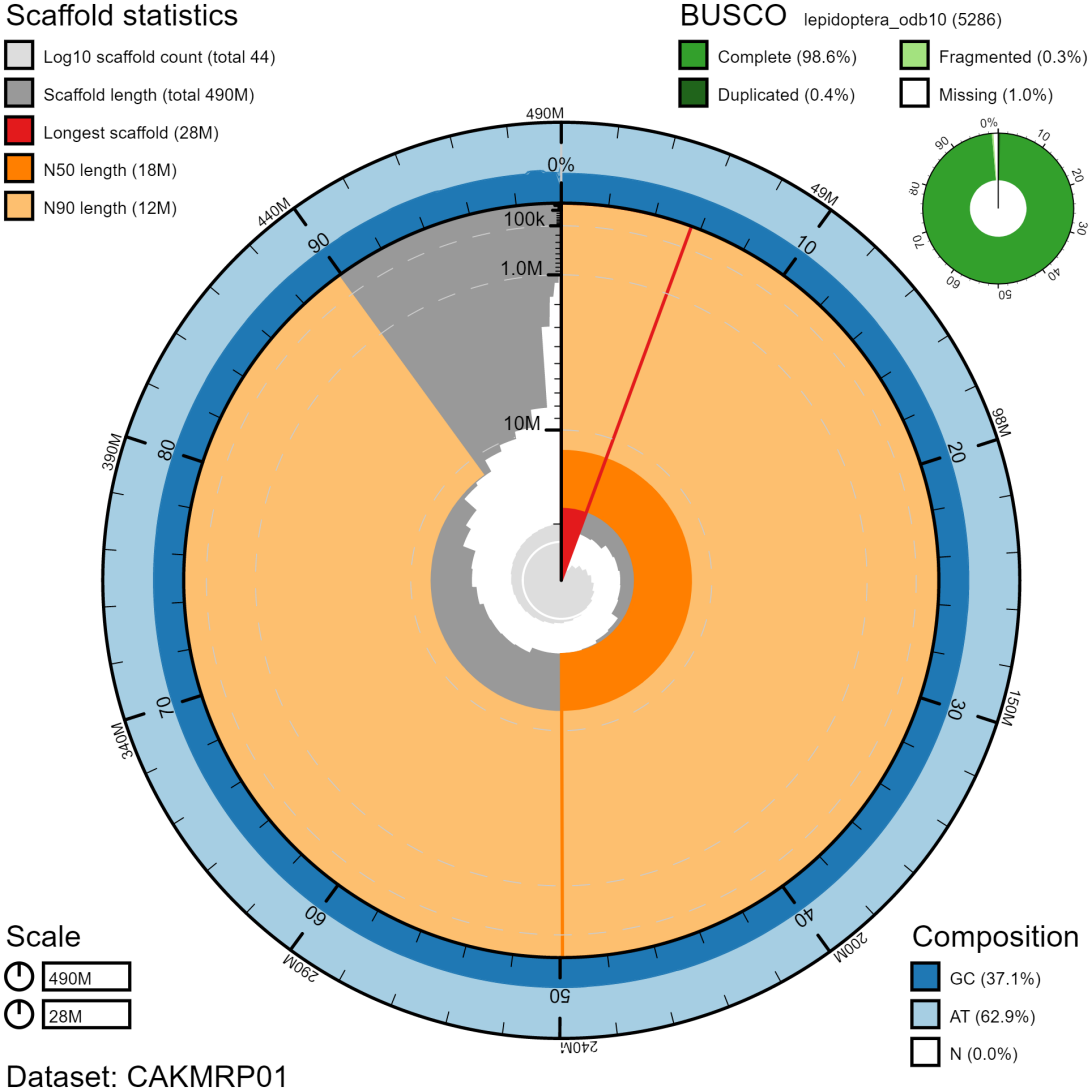
Genome assembly of *Lasiommata megera*, ilLasMege1.1: metrics. The BlobToolKit Snailplot shows N50 metrics and BUSCO gene completeness. The main plot is divided into 1,000 size-ordered bins around the circumference with each bin representing 0.1% of the 488,457,974 bp assembly. The distribution of chromosome lengths is shown in dark grey with the plot radius scaled to the longest chromosome present in the assembly (27,639,616 bp, shown in red). Orange and pale-orange arcs show the N50 and N90 chromosome lengths (17,836,388 and 11,819,885 bp), respectively. The pale grey spiral shows the cumulative chromosome count on a log scale with white scale lines showing successive orders of magnitude. The blue and pale-blue area around the outside of the plot shows the distribution of GC, AT and N percentages in the same bins as the inner plot. A summary of complete, fragmented, duplicated and missing BUSCO genes in the lepidoptera_odb10 set is shown in the top right. An interactive version of this figure is available at https://blobtoolkit.genomehubs.org/view/ilLasMege1.1/dataset/CAKMRP01/snail.

The final assembly has a total length of 488 Mb in 43 sequence scaffolds with a scaffold N50 of 17.8 Mb ( Table 1). The majority, 99.97%, of the assembly sequence was assigned to 30 chromosomal-level scaffolds, representing 28 autosomes (numbered by sequence length) and the W and Z sex chromosomes ( Figure 2– Figure 5; Table 2). The assembly has a BUSCO v5.3.2 ( Manni *et al.*, 2021) completeness of 98.6% (single 98.2%, duplicated 0.4%) using the lepidoptera_odb10 reference set (n=5,286). While not fully phased, the assembly deposited is of one haplotype. Contigs corresponding to the second haplotype have also been deposited.

**Table 1.** Genome data for *Lasiommata megera*, ilLasMege1.1.

*Project accession data*	
Assembly identifier	ilLasMege1.1
Species	*Lasiommata megera*
Specimen	ilLasMege1 (genome assembly); ilLasMege3 (Hi-C)
NCBI taxonomy ID	111917
BioProject	PRJEB48330
BioSample ID	SAMEA7523153
Isolate information	Female, whole organism (ilLasMege1); male, whole organism tissue (ilLasMege3)
*Raw data accessions*	
PacificBiosciences SEQUEL II	ERR7224284
10X Genomics Illumina	ERR7220443-ERR7220446
Hi-C Illumina	ERR7220447
*Genome assembly*	
Assembly accession	GCA_928268935.1
*Accession of alternate* *haplotype*	GCA_928267235.1
Span (Mb)	488
Number of contigs	59
Contig N50 length (Mb)	17.8
Number of scaffolds	43
Scaffold N50 length (Mb)	17.8
Longest scaffold (Mb)	20.7
BUSCO * genome score	C:98.6%[S:98.2%,D:0.4%], F:0.3%,M:1.0%,n:5,286

*BUSCO scores based on the lepidoptera_odb10 BUSCO set using v5.3.2. C= complete [S= single copy, D=duplicated], F=fragmented, M=missing, n=number of orthologues in comparison. A full set of BUSCO scores is available at https://blobtoolkit.genomehubs.org/view/ilLasMege1.1/dataset/CAKMRP01/busco.

**Figure 3.**
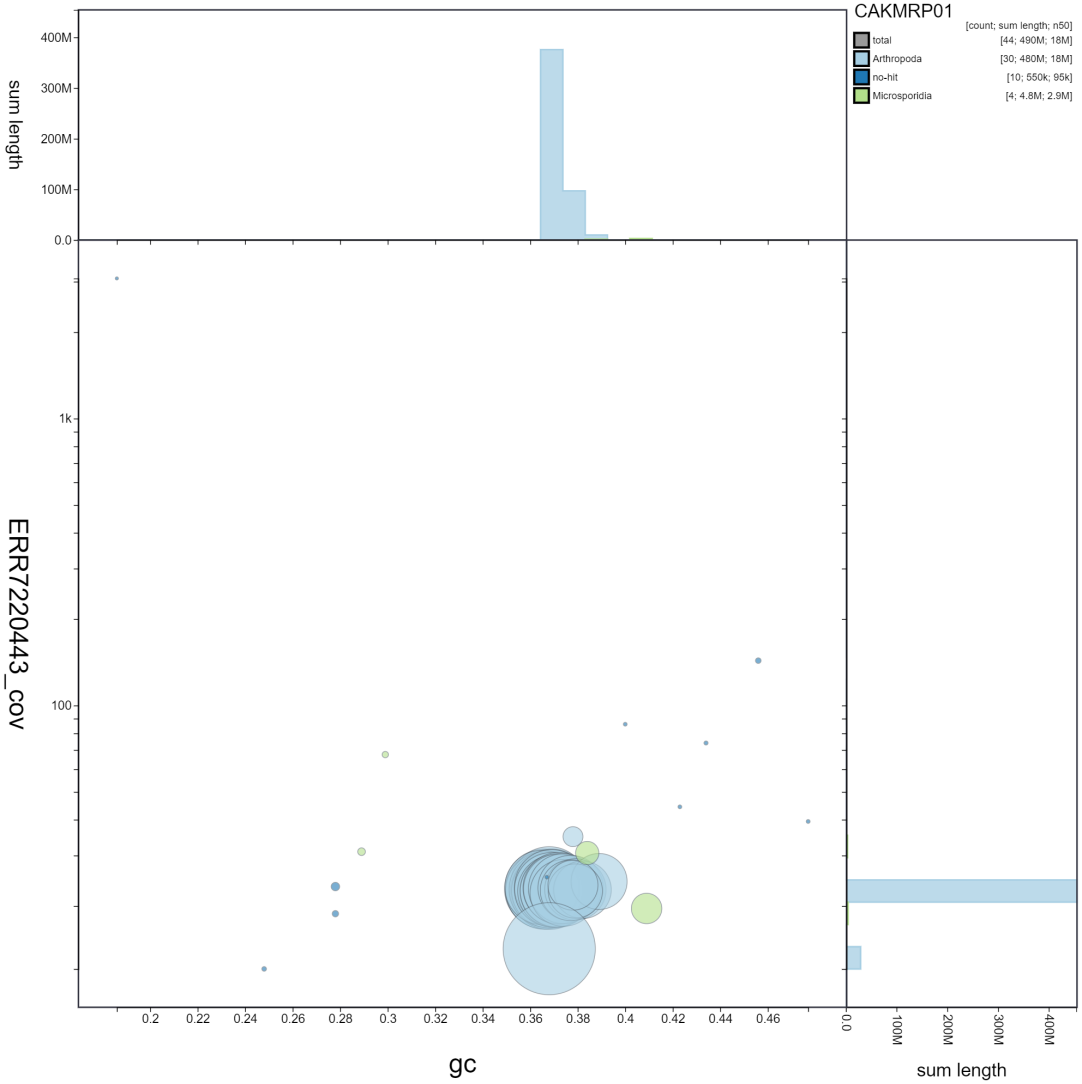
Genome assembly of *Lasiommata megera*, ilLasMege1.1: GC coverage. BlobToolKit GC-coverage plot. Scaffolds are coloured by phylum. Circles are sized in proportion to scaffold length. Histograms show the distribution of scaffold length sum along each axis. An interactive version of this figure is available at https://blobtoolkit.genomehubs.org/view/ilLasMege1.1/dataset/CAKMRP01/blob.

**Figure 4.**
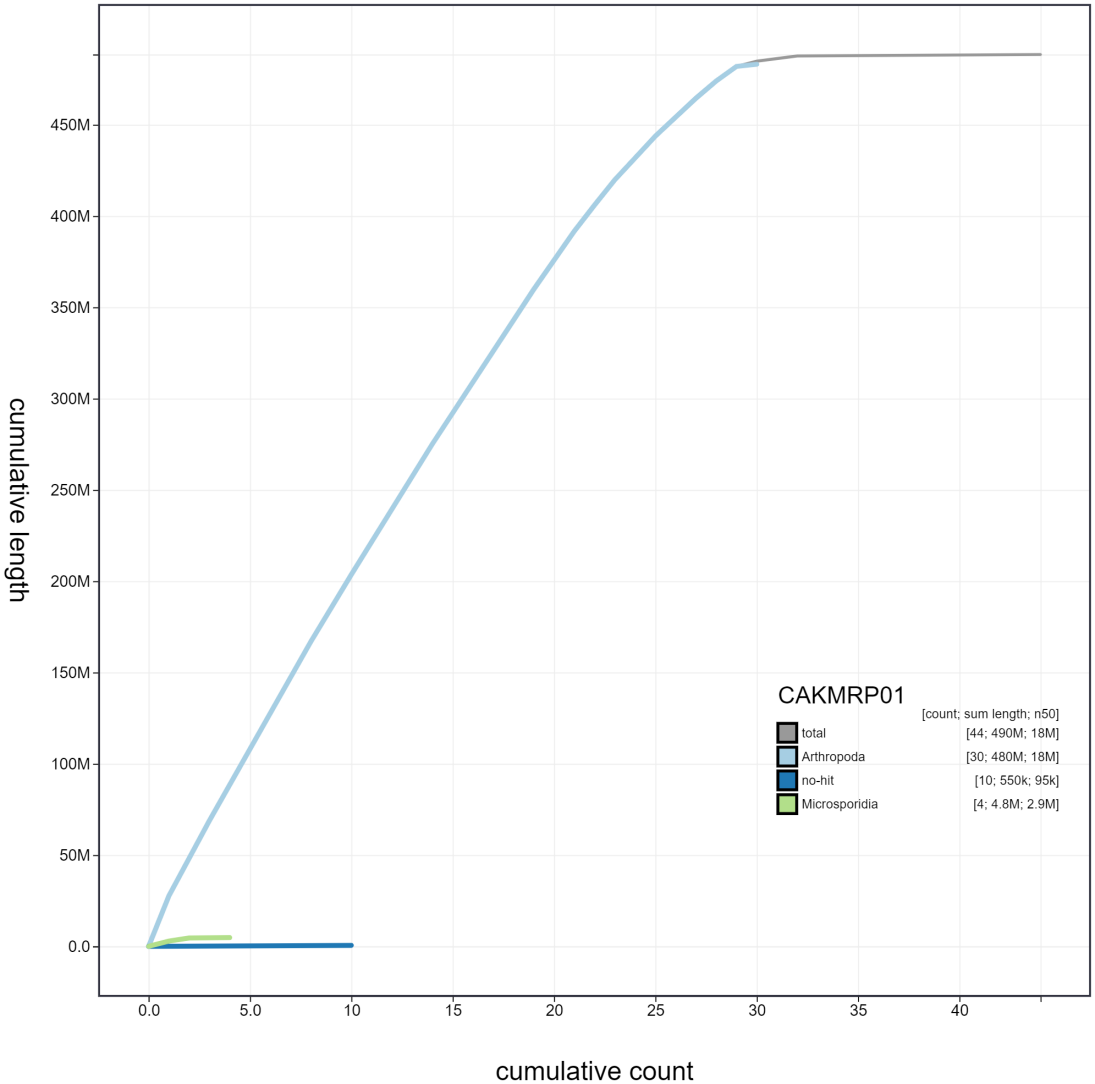
Genome assembly of *Lasiommata megera*, ilLasMege1.1: cumulative sequence. BlobToolKit cumulative sequence plot. The grey line shows cumulative length for all scaffolds. Coloured lines show cumulative lengths of scaffolds assigned to each phylum using the buscogenes taxrule. An interactive version of this figure is available at https://blobtoolkit.genomehubs.org/view/ilLasMege1.1/dataset/CAKMRP01/cumulative.

**Figure 5.**
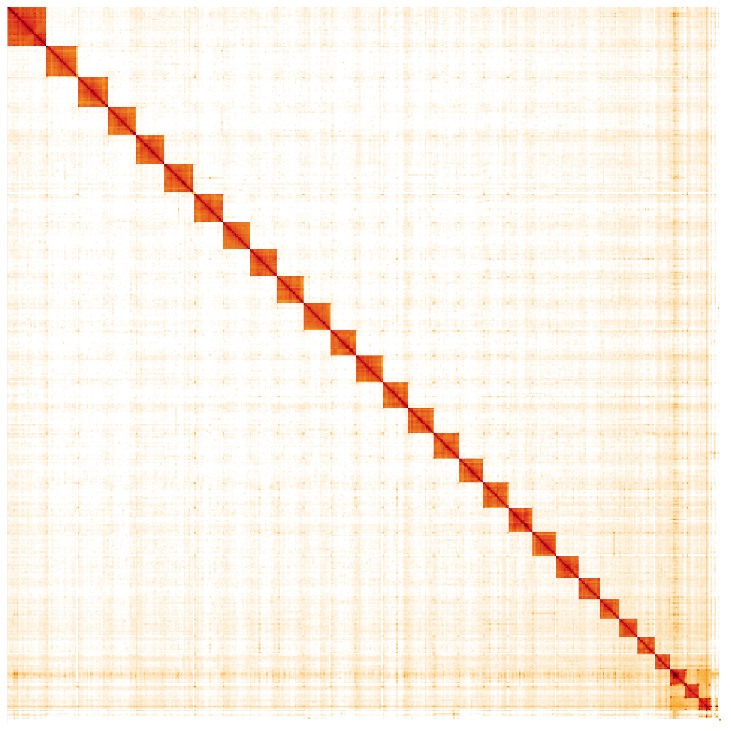
Genome assembly of *Lasiommata megera*, ilLasMege1.1: Hi-C contact map. Hi-C contact map of the ilLasMege1.1 assembly, visualised in HiGlass. Chromosomes are arranged in size order from left to right and top to bottom. The interactive Hi-C map can be viewed at https://genome-note-higlass.tol.sanger.ac.uk/l/?d=KdiGAPlYRgGFQxeB4jbmoA.

**Table 2.** Chromosomal pseudomolecules in the genome assembly of *Lasiommata megera*, ilLasMege1.1.

INSDC accession	Chromosome	Size (Mb)	GC%
OV743308.1	1	20.7	36.6
OV743309.1	2	20.53	36.9
OV743310.1	3	19.87	36.6
OV743311.1	4	19.7	36.8
OV743312.1	5	19.55	36.9
OV743313.1	6	19.5	36.6
OV743314.1	7	19.4	36.8
OV743315.1	8	18.45	36.7
OV743316.1	9	18.3	36.8
OV743317.1	10	18	36.9
OV743318.1	11	17.9	36.5
OV743319.1	12	17.84	36.9
OV743320.1	13	17.82	36.9
OV743321.1	14	17.19	37.1
OV743322.1	15	17.08	37.1
OV743323.1	16	16.99	37
OV743324.1	17	16.92	37.1
OV743325.1	18	16.47	37.2
OV743326.1	19	15.99	37.2
OV743327.1	20	15.84	37.4
OV743328.1	21	14.4	37.4
OV743329.1	22	13.76	37.7
OV743330.1	23	12.12	37.9
OV743331.1	24	11.82	37.7
OV743332.1	25	10.64	38.2
OV743333.1	26	10.07	38.9
OV743334.1	27	9.44	37.9
OV743335.1	28	7.99	37.8
OV743336.1	W	2.9	40.9
OV743307.1	Z	27.64	36.8
OV743337.1	MT	0.02	18.9
-	Unplaced	3.63	36.9

## Methods

### Sample acquisition and nucleic acid extraction

A single female *L. megera* specimen (ilLasMege1, genome assembly) was collected using a hand net from Aberlady Bay, Scotland, UK (latitude 56.019964, longitude -2.85808) by Konrad Lohse (University of Edinburgh). The specimen was identified by Konrad Lohse and snap-frozen in liquid nitrogen.

A single male *L. megera* specimen (ilLasMege3, Hi-C) was collected from the A1, East Linton, Scotland, UK (latitude 55.977161, longitude -2.667545) by Konrad Lohse (University of Edinburgh). The specimen was identified by Konrad Lohse and snap-frozen in liquid nitrogen.

DNA was extracted at the Scientific Operations Core, Wellcome Sanger Institute. The ilLasMege1 sample was weighed and dissected on dry ice. Whole organism tissue was disrupted by manual grinding with a disposable pestle. Fragment size analysis of 0.01–0.5 ng of DNA was then performed using an Agilent FemtoPulse. High molecular weight (HMW) DNA was extracted using the Qiagen MagAttract HMW DNA extraction kit. Low molecular weight DNA was removed from a 200-ng aliquot of extracted DNA using 0.8X AMpure XP purification kit prior to 10X Chromium sequencing; a minimum of 50 ng DNA was submitted for 10X sequencing. HMW DNA was sheared into an average fragment size between 12–20 kb in a Megaruptor 3 system with speed setting 30. Sheared DNA was purified by solid-phase reversible immobilisation using AMPure PB beads with a 1.8X ratio of beads to sample to remove the shorter fragments and concentrate the DNA sample. The concentration of the sheared and purified DNA was assessed using a Nanodrop spectrophotometer and Qubit Fluorometer and Qubit dsDNA High Sensitivity Assay kit. Fragment size distribution was evaluated by running the sample on the FemtoPulse system.

### Sequencing

Pacific Biosciences HiFi circular consensus and 10X Genomics Chromium read cloud sequencing libraries were constructed according to the manufacturers’ instructions. Sequencing was performed by the Scientific Operations core at the Wellcome Sanger Institute on Pacific Biosciences SEQUEL II (HiFi) and Illumina HiSeq (10X) instruments. Hi-C data were generated in the Tree of Life laboratory from whole organism tissue of ilLasMege3 using the Arima v2 kit and sequenced on a NovaSeq 6000 instrument.

### Genome assembly

Assembly was carried out with Hifiasm ( Cheng *et al.*, 2021); haplotypic duplication was identified and removed with purge_dups ( Guan *et al.*, 2020). One round of polishing was performed by aligning 10X Genomics read data to the assembly with longranger align, calling variants with freebayes ( Garrison & Marth, 2012). The assembly was then scaffolded with Hi-C data ( Rao *et al.*, 2014) using SALSA2 ( Ghurye *et al.*, 2019). The assembly was checked for contamination and corrected using the gEVAL system ( Chow *et al.*, 2016) as described previously ( Howe *et al.*, 2021). Manual curation ( Howe *et al.*, 2021) was performed using gEVAL, HiGlass ( Kerpedjiev *et al.*, 2018) and Pretext. The mitochondrial genome was assembled using MitoHiFi ( Uliano-Silva *et al.*, 2021), which performs annotation using MitoFinder ( Allio *et al.*, 2020). The genome was analysed and BUSCO scores generated within the BlobToolKit environment ( Challis *et al.*, 2020). Table 3 contains a list of all software tool versions used, where appropriate.

**Table 3.** Software tools used.

Software tool	Version	Source
Hifiasm	0.15.3	Cheng *et al.*, 2021
purge_dups	1.2.3	Guan *et al.*, 2020
SALSA2	2.2	Ghurye *et al.*, 2019
longranger align	2.2.2	https://support.10xgenomics.com/ genome-exome/software/pipelines/ latest/advanced/other-pipelines
freebayes	1.3.1-17- gaa2ace8	Garrison & Marth, 2012
MitoHiFi	2.0	Uliano-Silva *et al.*, 2021
HiGlass	1.11.6	Kerpedjiev *et al.*, 2018
PretextView	0.2.x	https://github.com/wtsi-hpag/ PretextView
BlobToolKit	3.2.7	Challis *et al.*, 2020

## Data availability

European Nucleotide Archive: Lasiommata megera (wall brown). Accession number PRJEB48330; https://identifiers.org/ena.embl/PRJEB48330.

The genome sequence is released openly for reuse. The *L. megera* genome sequencing initiative is part of the Darwin Tree of Life (DToL) project. All raw sequence data and the assembly have been deposited in INSDC databases. The genome will be annotated and presented through the Ensembl pipeline at the European Bioinformatics Institute. Raw data and assembly accession identifiers are reported in Table 1.

## Author information

Members of the Wellcome Sanger Institute Tree of Life programme are listed here: https://doi.org/10.5281/zenodo.6866293.

Members of Wellcome Sanger Institute Scientific Operations: DNA Pipelines collective are listed here: https://doi.org/10.5281/zenodo.5746904.

Members of the Tree of Life Core Informatics collective are listed here: https://doi.org/10.5281/zenodo.6125046.

Members of the Darwin Tree of Life Consortium are listed here: https://doi.org/10.5281/zenodo.5638618.
