# Supplementary material for: The genome sequence of the wall brown, Lasiommata megera (Linnaeus, 1767)
Source: Wellcome Open Res. 2022 Sep 12;7:230. [Version 1] doi: 10.12688/wellcomeopenres.18106.1 (PMC9971637; doi:10.12688/wellcomeopenres.18106.1)
Supplement: Peer review history

# Peer review: Reviewer response for version 1

**DOI:** 10.21956/wellcomeopenres.20077.r54914

**Published:** 2023-03-13

**Authors:** Yu-Shin Nai

**Affiliations:** 1 National Chung Hsing University,, Taichung, Taiwan

This report is about the whole genomic sequencing of an individual female *Lasiommata megera* (the wall brown; Arthropoda; Insecta; Lepidoptera; Nymphalidae). I have only two suggestions:

1. Is there any wet lab data provided (i.e. karyotyping of the butterfly) to validate the chromosomal level assembly?
2. Is there any microbe DNA fragment sequenced in the dataset? How does the microorganism tp be excluded? 

Are sufficient details of methods and materials provided to allow replication by others?

Yes

Is the rationale for creating the dataset(s) clearly described?

Yes

Are the datasets clearly presented in a useable and accessible format?

Yes

Are the protocols appropriate and is the work technically sound?

Yes

Reviewer Expertise:

My research is focusing on the genomics of insect pathogens, i.e., the transcriptome of entomopathogenic fungi (EPF) infected insect host, epigenetics of EPF and their regulation of gene expression and baculoviral genomics.

I confirm that I have read this submission and believe that I have an appropriate level of expertise to confirm that it is of an acceptable scientific standard.

# Peer review: Reviewer response for version 1

**DOI:** 10.21956/wellcomeopenres.20077.r54001

**Published:** 2023-03-13

**Authors:** Peter K Dearden

**Affiliations:** 1 Genomics Aotearoa and Department of Biochemistry, University of Otago, Dunedin, New Zealand

- This is a short article describing the production of a genome for a species of butterfly Lasiommata megera.
- The manuscript describes the rationale for sequencing this species' genome, identifying an appropriate specimen, and proper documentation of that specimen.
- The methods of DNA extraction, DNA sequencing and assembly are well described and explained, and the manuscript provides excellent information as to the approaches taken.
- The genome produce is high quality and publically available.
- My only concern with this work is that the script used by the team do not seem to be available, or if they are available, not shown. I would hope that a github (or similar) archive might be available to help readers re-run the analysis if they wish.
- Other than this minor point, this is an excellent addition to our datasets of insect genomes.

Are sufficient details of methods and materials provided to allow replication by others?

Partly

Is the rationale for creating the dataset(s) clearly described?

Yes

Are the datasets clearly presented in a useable and accessible format?

Yes

Are the protocols appropriate and is the work technically sound?

Yes

Reviewer Expertise:

Insect genomics, evolution and development

I confirm that I have read this submission and believe that I have an appropriate level of expertise to confirm that it is of an acceptable scientific standard.

# Peer review: Reviewer response for version 1

**DOI:** 10.21956/wellcomeopenres.20077.r54921

**Published:** 2023-02-27

**Authors:** Jérémy Gauthier

**Affiliations:** 1 Muséum cantonal des sciences naturelles, Lausanne, Switzerland

In this paper, the authors provide the chromosome-scale assembly of the genome of the wall brown, Lasiommata megera. 

 The process of genome assembly and quality assessment is broadly within current standards although additional statistics such as base level accuracy (QV) could have been estimated.

 In the first sentence of the paragraph "Genome assembly", it should be specified that the main assembly with Hifiasm was performed on PacBio data.

 This new genome is in line with the current process of generating new reference genomes for a wide variety of species and thus provides a valuable tool for further analysis of this species.

Are sufficient details of methods and materials provided to allow replication by others?

Yes

Is the rationale for creating the dataset(s) clearly described?

Yes

Are the datasets clearly presented in a useable and accessible format?

Yes

Are the protocols appropriate and is the work technically sound?

Yes

Reviewer Expertise:

Populations genomics, Museomics

I confirm that I have read this submission and believe that I have an appropriate level of expertise to confirm that it is of an acceptable scientific standard.

# Peer review: Reviewer response for version 1

**DOI:** 10.21956/wellcomeopenres.20077.r53992

**Published:** 2023-01-16

**Authors:** Christopher W Wheat

**Affiliations:** 1 Stockholm University, Stockholm, Sweden

This is a standard genomic report for a common field butterfly, reporting a chromosomal level assembly for a female, therefore including the Z and W chromosomes. Standard reporting of the methods and quality of the assembly are contained, reporting that there is a very high quality assembly that has been sufficiently resolved to a haploid state, albeit without phasing though the alternative haplotypes are provided. No annotation is provided. The reported 28 autosomes are consistent with the reported haploid chromosomal count of *L. menava*, from the karyology literature from Robinson, 1971.

Are sufficient details of methods and materials provided to allow replication by others?

Yes

Is the rationale for creating the dataset(s) clearly described?

Yes

Are the datasets clearly presented in a useable and accessible format?

Yes

Are the protocols appropriate and is the work technically sound?

Yes

Reviewer Expertise:

butterfly functional genomics

I confirm that I have read this submission and believe that I have an appropriate level of expertise to confirm that it is of an acceptable scientific standard.
